# The role of clinical medical physicists in the future: Quality, safety, technology implementation, and enhanced direct patient care

**DOI:** 10.1002/acm2.12619

**Published:** 2019-05-17

**Authors:** Brian Wang, Gerald White

**Affiliations:** ^1^ Yale University School of Medicine New Haven CT USA; ^2^ Colorado Associates in Medical Physics Colorado Springs CO USA

A few months ago, this journal published an editorial by Pawlicki and Mundt titled “Continued emphasis on quality and safety jeopardizes clinical medical physics careers in radiation oncology: What can be done about it?”^1^. We were surprised by the position of the authors (“…the continued emphasis on quality and safety is likely a threat to the long‐term viability of clinical medical physicists in radiation oncology.”) Both authors have been staunch and effective advocates for quality and safety in radiation oncology. They are both among the authors of the book “Quality and Safety in Radiotherapy” and have published extensively on this topic.^2^ What could cause them to think clinical medical physicists should not focus on quality and safety? The answer is found deep in the editorial, following a summary of the authors' impression of the routine tasks performed by oncology medical physicists as well as the associated value. We begin by offering an alternative understanding of the clinical contributions of the oncology medical physicist and then address the proposals of Pawlicki and Mundt for a revised path for the future of our profession. We find much to agree with in their editorial, but believe the substance of the arguments to be fundamentally flawed, without the likelihood of either adoption, or success if adopted.

Let us look at some of the activities of clinical medical physicists in radiation oncology, following the outline of Pawlicki and Mundt. Responsibilities such as equipment calibration and quality assurance (QA), patient‐specific QA, treatment plan checks, weekly chart checks, and patient‐specific measurements are characterized as “routine” and “checking” activities not generally requiring the expertise of the medical physicist. We disagree. Tasks performed over and over, day after day are correctly described as “routine,” a description that refers to their temporal repetition but should not be taken to denigrate the intellectual effort and expertise that is used for each unique instance. Medical physicists should not be at risk for replacement by individuals with more limited training if we, at each of these “routine” endeavors, bring to bear the full spectrum of our training and expertise, unrivaled by anyone else on the radiation oncology team. After the fact, one could identify particular tasks or steps for a particular patient that could have been undertaken by another individual on the team, but it is not possible to identify, a priori, all the possible failure modes that a properly educated and trained medical physicist might identify in a sequence of technical or clinical reviews. It is the ability of the medical physicist to integrate the physical, anatomic, and clinical aspects of each individual patient treatment sequence that brings value to the process.

We agree with Pawlicki and Mundt that automation of QA, increased reliability of equipment, etc., will certainly make much of the work we currently perform less time‐consuming or even unnecessary. This is not a new phenomenon in our profession. One of us can remember digitizing patient contours (taken by solder wire) by typing in hundreds of (x,y) coordinates into a teletype console to be used by a remote radiation therapy planning system. Current technologies allow us to enter spatial (and density) information with millions more data points in a few seconds. Do we now have hours left over with nothing to do? No, we spend even more time with tasks such as preparing stereotactic isodose plans, consulting with radiation oncologists, neurosurgeons, pulmonologists, and others on the details of the proposed treatments and then implementing them on the treatment machines. Earlier effort at performing the dosimetric plan verification associated with intensity modulated radiation therapy (IMRT) involved a very lengthy process using film for each of 6–9 fields, scanning densitometers, H&D corrections, rigid processor quality control, and image analysis. Fast‐forward to today, we have diode‐ and EPID‐based techniques, but our profession continues to advance in value. An increase in complexity and automation often leads to an increase in the need for professional expertise for the big picture, not a decrease.^3^

We will need to continue to adapt “routine” QA to meet changing technology. New techniques for new imaging, planning, and delivery systems will be necessary and will require the efforts of medical physicists to design, test, and implement these procedures. While others will do some tests, we will perform some ourselves and personally review and analyze the entire spectrum of testing, assisted by automated techniques. QA tasks, and benchmarks for existing technology will be adjusted in light of current design and clinical implementation.^4^

Some additional, specific examples:

One of the routine duties for clinical medical physicists is to check patient‐specific charts. This includes both plan review prior to the first treatment and ongoing weekly checks. We believe these activities are very important for the quality and safety of patient care. On many occasions, physicists could find issues with the treatment plan such as missing couch or omitting density overwrite for contrast agent. Such catches can certainly improve the quality of the treatment plan with more accurate dose calculations. In less frequent cases, physicists may detect even bigger errors like wrong image set for planning, which otherwise could jeopardize the patient's safety. These independent plan reviews are critical to the quality and safety of patient care. Literature has shown that many potential errors are caught during the initial and ongoing chart review by clinical medical physicists.^5^ Some may argue that these plan review activities can be replaced by automation and artificial intelligence (AI), and that the role of clinical medical physicists therefore becomes less important for the quality and safety of patient care. Indeed, automation and AI are increasingly adopted in radiation oncology for tasks such as contouring, autoplanning, and plan checks. We do not believe, however, that automation would replace clinical medical physicists even for some of the simple tasks that computers can perform. Oftentimes, the task needs to be customized and modified by clinical medical physicists. After all, every patient is unique and a generic algorithm may not provide a one‐size‐fits‐all solution. More importantly, when potential issues are identified for a specific plan, it is clinical medical physicists rather than computers who communicate with other team members such as physicians, therapists and dosimetrists to reach a clinically acceptable solution.

Clinical medical physicists play a very important role when a treatment machine is down or has issues. Physicists need to discuss with service engineers to diagnose the problem and develop a repair plan. QA procedures are often required before the machine is released back for patient treatments and they must be performed by qualified clinical medical physicists after the repair. Some jurisdictions require the specific approval of the medical physicist post‐repair prior to returning the linac to clinical use. The degree of the physicist's involvement could vary depending on the competence of the service engineers. Regardless, a proactive and dedicated physicist can speed up the process for a faster return of the machine for patient care. Even though clinical medical physicists are not trained to repair machines, they can help identify the cause of the issue by their analytical skills and familiarity with the machine from routine QA. Another important role of clinical medical physicists during machine down time is to communicate the progress to physicians, therapists and dosimetrists, which can reduce the anxiety of patients and the whole cancer care team.

Another routine duty of clinical medical physicists is to participate in stereotactic body radiotherapy (SBRT) and stereotactic radiosurgery (SRS) setup and treatments. For simple cases, physicists verify the correct isocenter is used and the correct image guidance radiation therapy (IGRT) shifts are implemented, which is critical to the safety of treatments for these high‐dose procedures. For complicated cases, physicists evaluate the IGRT images and make recommendations to the physician colleagues. This requires their technical assessment of the motion management, knowledge of limitations of various imaging systems including the initial simulation computed tomography (CT) scan and cone beam CT (CBCT). One of us can testify that physicist's involvement is essential for gated SBRT treatments of liver cancer using the Calypso technology.^6^ Many steps are required to interact among the various control systems and the clinical medical physicist is essentially the “orchestra director” for the entire treatment delivery team.

As to other, clinically based changes to the practice of medical physics, we agree wholeheartedly with Pawlicki and Mundt that “Physicists think differently than medically trained healthcare professionals such as radiation oncologists or nurses.” (It is this proclivity for a different mode of thinking and possession of a different set of technical and scientific skills that brings value to the medical physics endeavor in the clinic.) They follow this thought with the unsupported assertion that “Unfortunately, the physicist's unique perspective is not being utilized because most of their time is spent checking the work of others.” In our experience, that is not the case. Even those activities that have a “checking” component, such as isodose plan review or weekly chart checks, also involve an analysis of the application of technology to the care process for an individual patient. We also agree that patients will benefit from increased patient contact and interaction. Future success of the medical physics profession will depend on this. While the logistics and practicality of assigning each patient a single medical physicist for the duration of their treatment seem both daunting and of dubious value, having the medical physicist interact directly with patients on a daily basis is common in our practice experience, particularly in brachytherapy, stereotactic procedures, isotope therapy, and the very common occurrence of solving set‐up problems in the treatment room. One of us has, on more than one occasion in a social setting, encountered a former patient and been introduced to others as “my medical physicist.”

We strongly support the call for increased training in clinical areas for medical physicists. Pawlicki and Mundt raise the possibility of medical physicists performing target volume delineation — a task transfer that is unlikely to benefit patients absent a multiyear addition to the medical physics training process to include serious preparation in anatomy as well as in physiology, pathology, general oncology, molecular, magnetic and radiographic imaging, medical and surgical oncology, chemical and genetic markers… the list goes on. They make the further suggestion that medical physics could engage in treatment plan approval; but we note that the medical physicist prior to administration must approve every treatment plan in our clinics. We see no patient benefit to removing the physician from the approval process.

Radiation oncology is a technology‐driven specialty and the clinical medical physicist is crucial to implementing new technologies for patient care. Over the past three decades, several new technologies (e.g., IMRT, IGRT, SBRT, proton therapy) have emerged and have become widely adopted in radiation oncology. It is worth to note that the need for clinical medical physicists has increased tremendously, for example, the number of American Association of Physicists in Medicine (AAPM) members has tripled during the same period of time.^7^ Today, more new technologies are either emerging or already available to radiation oncology such as MR‐Linac, FLASH, modulated brachytherapy. Clinical medical physicists have plenty of opportunities to use their training and skill set to implement these techniques for patient care.

We look forward to the implementation of a different, more clinically oriented training program for medical physicists that will lead to an increase in the direct contact between the patient and the medical physicist. Many of us are experiencing this in our clinics today and are hopeful that proliferation of that practice pattern will continue. We see no reason to denigrate the importance of the quality and safety work by the medical physicist to achieve this goal.

## CONFLICT OF INTEREST

None declared.
